# Trips for Health and Pleasure

**Published:** 1906-12-22

**Authors:** 


### TRIPS FOR HEALTH AND PLEASURE.

### TO BUENOS AYRES IN A ROYAL MAIL LINER.

This voyage has one great merit, namely, that after Cape Finisterre has been passed the weather is nearly always favourable; and even when crossing the Bay it is more likely than not that a fairly smooth sea will be met with. Although this statement is contrary to the generally accepted belief of most landsmen, it is nevertheless true, as the present writer can testify after numerous passages. The invalid will find that the temperature experienced on the voyage to South America is agreeable during the majority of the twelve months of the year; but, of course, the hottest months are December and January, and the coldest are July and August, so that if his condition require it the invalid should avoid these months.

After leaving Southampton the steamer touches at Cherbourg to take up passengers; but there is no time to land, and in half an hour or so the vessel goes on her journey across the Bay to Vigo. The distance from Ushant to Finisterre is about 380 miles. It is this distance that is known as crossing the Bay; but the vessel is really outside the Bay all the time, and with good luck and a good steamer it will be run in 24 hours. After leaving the Spanish town of Vigo, the Portuguese town of Leixœs (pronounced Leyshones) is reached next day, and the day following the vessel anchors in the Tagus opposite Lisbon. When Madeira is one of the ports called at, which it is once in three weeks, it is reached in about 36 hours from Lisbon, or about five days from Southampton. In eight days the Cape Verd Islands will be sighted, and a call made at St. Vincent. Five days afterwards Pernambuco will be reached. Then next day Bahia, and in three days from that port Rio Janeiro, and the day after Santos. Three days after leaving Santos the traveller will be in Monte Video, and within 24 hours Buenos Ayres. The voyage, therefore, is far from monotonous; and it is free from these long weeks at sea when the vessel seems alone in a world of water, and which to many invalids are so trying. There is time to land at nearly all the ports we have named, although in some of them not much can be done between the arrival and departure of the steamer; but the passenger can stay practically as long as he likes at any of the ports, going on by another of the company's vessels or returning to England by any homeward-bound one.

In Madeira the hotel accommodation is very good, and not extravagant. The company runs trips to this Island during the summer months for £21, including hotel expenses, and as this trip works out at about £1 a day for everything necessary it is not at all excessive for a first-class steamer. At other times of the year the return fare would seem to be about £23 14s., and this for a voyage of ten or eleven days does seem dear; and is the more remarkable as the return fare to Vigo is only £9 15s. and to Lisbon and back only £12; but there may be some good, if occult, reason for the difference.

At Pernambuco hotel accommodation may be obtained from 10s. a day upwards. At Bahia the rate is about the same, and there are both English and German clubs there.

Rio Janeiro, the capital of Brazil, is said to have the finest harbour in the world. There is in the town a considerable English colony, an English church, several good hotels, and at least one English medical man. Santos has the reputation of being an extremely healthy place, with the purest drinking water, which is brought to it from the hills ten miles off. Santos is only 34 miles from Sao Paulo, which town has 250,000 inhabitants. Monte Video, the capital of Uruguay, is blessed with one of the finest climates in the world. It possesses good hotels, and there is an English club; but we believe the English residents are not very numerous; still they are sufficient in number to have an English church and chaplain, and several English physicians practise there.

Buenos Ayres, the capital of the Argentine Republic, and the goal of this voyage, contains between eight hundred thousand and one million inhabitants. It is an old town founded nearly four hundred years ago. Needless to say there are plenty of hotels and restaurants, and a dozen or more English medical men live in the city.

The voyage which we have outlined would take about seven weeks if the passenger returned in the same steamer he went out by, and the seven weeks would allow him about one week in Buenos Ayres. The return fare is £62, and hotel expenses must be added to this. To Monte Video the fare is the same. To Rio and to Bahia £9 less, and to Pernambuco £12 less. The above are the lowest fares, and some cabins are charged an additional £7, and some of the steamers have cabines-de-luxe at much higher figures. It may, however, be assumed that for an invalid the 50-days' trip would cost him at least £80; but the benefit derivable from such a voyage might be very great, and more than worth the money to the man who can reasonably afford it.

Some of the vessels in this service are the finest in the Royal Mail Fleet, and run up to 10,000 tons.

Return tickets to Buenos Ayres are available to return by the West India branch of the Royal Mail line, the traveller going overland from Buenos Ayres to Valparaiso, and then steaming along the coast to Panama and joining the other steamer at Colon. Or he could go by the Straits of Magellan, and thus almost circumnavigate South America. In the former case the fare would be £101, and in the latter it would be £112.

The Chelsea Hospital for Women has received £21 from Mr. Simon Symons to its fund to maintain its twelve years' record of "freedom from debt." This fund exceeds £60, of which £10 10s. constitute new annual subscriptions.

